# Organic Material on Ceres: Insights from Visible and Infrared Space Observations

**DOI:** 10.3390/life11010009

**Published:** 2020-12-24

**Authors:** Andrea Raponi, Maria Cristina De Sanctis, Filippo Giacomo Carrozzo, Mauro Ciarniello, Batiste Rousseau, Marco Ferrari, Eleonora Ammannito, Simone De Angelis, Vassilissa Vinogradoff, Julie C. Castillo-Rogez, Federico Tosi, Alessandro Frigeri, Michelangelo Formisano, Francesca Zambon, Carol A. Raymond, Christopher T. Russell

**Affiliations:** 1 Istituto Nazionale di Astrofisica–Istituto di Astrofisica e Planetologia Spaziali, 00133 Rome, Italy; Mariacristina.desanctis@inaf.it (M.C.D.S.); filippo.carrozzo@inaf.it (F.G.C.); mauro.ciarniello@inaf.it (M.C.); batiste.rousseau@inaf.it (B.R.); marco.ferrari@inaf.it (M.F.); simone.deangelis@inaf.it (S.D.A.); federico.tosi@inaf.it (F.T.); alessandro.frigeri@inaf.it (A.F.); michelangelo.formisano@inaf.it (M.F.); Francesca.zambon@inaf.it (F.Z.); 2 Agenzia Spaziale Italiana, 00133 Rome, Italy; eleonora.ammannito@asi.it; 3 Physique des Interactions Ioniques et Moléculaires, PIIM, Université d’Aix-Marseille, 13013 Marseille, France; vassilissa.vinogradoff@univ-amu.fr; 4 Jet Propulsion Laboratory, California Institute of Technology, Pasadena, CA 91109, USA; Julie.C.Castillo@jpl.nasa.gov (J.C.C.-R.); carol.a.raymond@jpl.nasa.gov (C.A.R.); 5 Earth Planetary and Space Sciences, University of California, Los Angeles, CA 90095, USA; ctrussel@epss.ucla.edu

**Keywords:** astrobiology, organic material, Ceres, small bodies

## Abstract

The NASA/Dawn mission has acquired unprecedented measurements of the surface of the dwarf planet Ceres, the composition of which is a mixture of ultra-carbonaceous material, phyllosilicates, carbonates, organics, Fe-oxides, and volatiles as determined by remote sensing instruments including the VIR imaging spectrometer. We performed a refined analysis merging visible and infrared observations of Ceres’ surface for the first time. The overall shape of the combined spectrum suggests another type of silicate not previously considered, and we confirmed a large abundance of carbon material. More importantly, by analyzing the local spectra of the organic-rich region of the Ernutet crater, we identified a reddening in the visible range, strongly correlated to the aliphatic signature at 3.4 µm. Similar reddening was found in the bright material making up Cerealia Facula in the Occator crater. This implies that organic material might be present in the source of the faculae, where brines and organics are mixed in an environment that may be favorable for prebiotic chemistry.

## 1. Introduction

Ceres is the largest object in the asteroid belt. Because of its size (~950 km in diameter) and its spheroidal shape, it has been classified as a dwarf planet. Spectrophotometric ground observations of Ceres have revealed a complex mineralogy from the absorption band depths and positions along the spectrum of the solar flux reflected by its surface. The low albedo and the relatively flat visible/near-IR (0.4–2.5 µm) spectrum suggest the presence of a dark component like magnetite [1,2]. Absorption bands at 3.3–3.4 µm and 3.8–4.0 µm have been interpreted by carbonates like dolomite, calcite, and magnesite, which is consistent with observations in the mid-infrared [1,2]. A narrow absorption at 3.06 µm has been attributed to different species, like ice frost [3], NH_4_-bearing minerals [4], irradiated organics and crystalline water ice [5], cronstedtite [6], and brucite [1]. 

The Dawn NASA mission represents a breakthrough for the investigation of Ceres. Dawn’s payload includes a visible and infrared mapping spectrometer (VIR) [7], a framing camera (FC) with seven wavelength filters [8] and a gamma ray and neutron detector (GRaND) [9]. The three instruments were complementary in inferring the surface properties of Ceres.

The VIR imaging spectrometer is composed of two channels: the VIS channel covers the spectral range 0.25–1 µm, with a spectral sampling of ~2 nm, and the IR channel covers the spectral range 1–5 µm, with a spectral sampling of ~10 nm. The VIR obtained spectra of Ceres with unprecedented quality and quantity for an icy body, without any limitations due to atmospheric absorptions (which hide the range of 2.5–2.9 microns), and at a resolution of about a few kilometers to tens of meters. 

The first finding from the VIR-IR channel shows a global surface composed of a dark component (carbon and/or magnetite), and Mg-carbonates such as dolomite, confirming some of the previous studies based on ground observations. The discovery of a strong absorption at 2.7 µm (confirmed by data from the satellite AKARI [10]) was assigned to Mg-phyllosilicates such as antigorite, and a better definition of the 3.06-µm absorption was attributed to NH_4_-phyllosilicates [11]. 

Then, the GRaND instrument provided new constraints. Based on this elemental data, in particular the abundance of C, H, K, and Fe, [12,13] it was established that the dark material that makes up most of Ceres’ surface composition should be rich in carbon and it resembles carbonaceous chondrite material [14,15] which is associated with a thick volatile-rich crust, thus suggesting a large and diffuse presence of organic material.

Recent improvement in the VIS channel calibration [16,17] allows for extending the range of the VIR instrument to shorter wavelengths (0.25–1.0 µm). Here, we further refine the calibration of the IR channel following Rousseau et al. [16,17]. The resulting VIR data in the visible range, together with the refined calibration in the IR channel provide new constraints on Ceres mineralogy. Using this new capability, we performed spectral analysis and modelling to infer further details on the composition of the dwarf planet. In the present work, we focus on the average spectrum of Ceres. We also revise local spectra from the Ernutet and Occator crater regions, where the VIR-IR instrument detected clear signatures of aliphatic organics [18] and a mixture of dry and hydrated salts, respectively, with the possible presence of organics [19]. 

## 2. Average Ceres Spectrum 

### 2.1. Calibration Refinement 

We collected calibrated VIR data (available through the Planetary Data System (PDS) online data archive at: https://sbn.psi.edu/pds/resource/dawn/dwncvirL1.html). The data investigated in this work were acquired during different phases of the Dawn mission at Ceres, and they provide almost complete coverage of Ceres’ surface. The calibrated dataset was further refined with the procedure described in Carrozzo et al. [20] and Rousseau et al. [16], in order to correct for the odd-even effect and systematic artifacts [20], and spurious spectral variations due to the detector temperature in the VIS channel [16]. Data were then photometrically corrected to standard viewing geometry (incidence angle = 30°, emission angle = 0°, phase angle = 30°) by means of Hapke modeling [21,22] according to the photometric parameters derived by Ciarniello et al. [23]. Moreover, we applied correction factors derived from ground-based observations to correct for fictitious slopes on spectra of both VIR channels. These correction factors were first derived by Carrozzo et al. [20]. In the present work, we used the updated version for the VIS channel derived by Rousseau et al. [17], and an updated version for the IR channel, here proposed, which was derived following the same procedure of Rousseau et al. [17]. Here we summarize the main steps to produce the ground correction.

1. We collected ground observations of Ceres, which are mutually consistent in the spectral range, where they overlap [2,24,25,26,27,28,29,30].
2. For each ground full-disk observation (point 1), bidirectional reflectance was converted to standard viewing geometry (incidence angle = 30°, emission angle = 0°, phase angle = 30°) by means of Hapke modeling [22] according to the photometric parameters derived by [23].
3. Based on the ground-based spectra (point 2), we calculated a smooth average spectrum that covers the whole spectral range of the infrared channel of the VIR spectrometer.
4. We calculated the average spectrum of VIR-IR calibrated data, after artifact and photometric correction, as described above.
5. We calculated the ratio between the average spectrum from ground observations (point 3) with the average spectrum obtained from VIR data (point 4). This ratio spectrum is used as a multiplicative correction factor on every single VIR spectrum.

**Figure 1:**
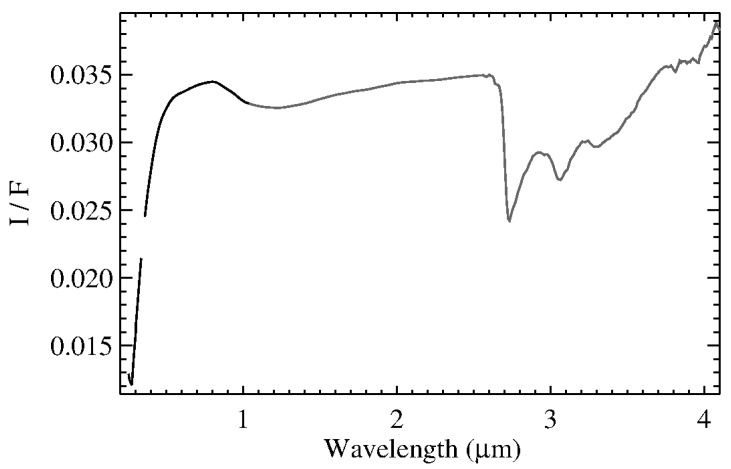
Average Ceres spectrum. The VIS channel (black) and IR channel (gray) have been merged to obtain a unique spectrum. The spectral range longward of 4.1 µm (not shown here) is affected by high noise level after the subtraction of the thermal emission.

Merging the global average spectrum obtained in the VIS channel [17] with the average IR spectrum derived here, we obtained a single global average spectrum of Ceres in the range of 0.25–5.0 µm. Then, we removed the thermal emission affecting the spectrum longward of 3.5 µm, as discussed in Section 2.2 (Figure 1).

### 2.2. Spectral Modeling

To obtain information on the abundance of the surface minerals, we used a quantitative spectral analysis of the composition using Hapke’s radiative transfer model [21,22]. Similar modeling has been described by De Sanctis et al. [11], Marchi et al. [14], and Raponi et al. [31]. The whole formulation of the bidirectional reflectance (r) is:

r = (SSA/4π) μ_0_/(μ+ μ_0_) × K [B_SH_ p (g) + H (SSA, μ/K) H (SSA, μ_0_/K) − 1] × S (i, e, g, θ) B_CB_

where i, e, g are the incidence, emission, and phase angles, respectively, and μ_0_, μ are the cosines of the incidence and emission angles. These parameters come from the shape model and position of the spacecraft at the time of observation. The parameters that contain most of the spectral information are the single scattering albedo (SSA), and the related Ambartsumian–Chandrasekhar functions H (SSA, μ/K), which describe the multiple scattering components. Other parameters that describe the photometric behavior as a function of the viewing geometry are: the single particle phase function p (g); the shadow hiding opposition effect B_SH_ (g); the coherent back-scattering opposition effect B_CB_ (g); the shadow function modeling large-scale roughness S (i, e, g, θ), with θ being the average surface slope; and the porosity parameter, K linked to the filling factor [22]. These photometric parameters are fixed after Ciarniello et al. [23], who defined the average scattering properties of Ceres’ regolith. The spectral properties are mainly affected by the SSA. The latter have been modeled for intimate mixing between different minerals, which implies that the particles of the endmember materials are in contact with each other and are all involved in the scattering of a single photon. The SSA of each mineral is defined starting from their grain size and their optical constants, as described in [18]. The optical constants are derived from laboratory measurements (Table 1) with the method described by Carli et al. [32]. The average SSA of the regolith is defined through the weight p_i_, which is the cross section of all grains of the *i*th mineral as a fraction of the total area. To simplify the model and minimize the number of free parameters, we assigned the same grain size to all endmembers. In this way, the relative cross section of each endmember is identical to the relative volumetric abundance. The best result is obtained by comparison of the model with the measured spectra by applying a curve-fitting procedure. The free model parameters to be retrieved are: (i) abundance of the endmembers and the grain size, which define the SSA; (ii) a multiplicative constant and additional slope to the SSA of the model in order to account for uncertainties in the radiometric and photometric accuracies, as well as other unknown neutral minerals making up the surface; and (iii) temperature T and beaming function ∧ [33]. The latter is multiplied by the directional emissivity ε_d_ [22] to give the effective emissivity (ε_eff_). The directional emissivity is a function of the SSA (wavelength-dependent) and the emission angle. The beaming function accounts for the fact that a macroscopically rough surface does not emit radiation in the same way as a macroscopically flat surface, since there are a variety of local normals across the surface [33]. Interpretation of the modeled thermal emission is outside the scope of this work. The total radiance is modeled by accounting for both the contribution of the reflected sunlight, and the thermal emission:

Rad = r × F_ʘ_/D^2^ + ε_eff_ × B(λ, T)

where r is the Hapke bidirectional reflectance, F_ʘ_ is the solar irradiance at 1 AU, D is the heliocentric distance (in AU), ε_eff_ is the effective emissivity, and B(λ, T) is the Planck function. Thus, the estimation of the thermal emission is done simultaneously with the reflectance modeling to yield a consistent result between these two contributions to the total signal measured. Here, we show the measured spectra after the subtraction of thermal emission to isolate the contribution of the reflectance spectra.

We discarded the spectral range shortward of 0.36 µm from the analysis because it has a high level of uncertainty due to the low sensitivity of the detector, and the spectral range longward of 4.1 µm, because it is affected by a high noise level after the subtraction of the thermal emission.

The SSA was modeled starting with minerals that have already been discussed in De Sanctis et al. [11,34] (see Table 1). However, to account for the spectral shape in the VIS channel, we added the spectrum of lizardite measured by Hiroi and Zolensky [35] who heated the sample in a vacuum at 600 °C to remove water contaminating the sample. Such a spectrum presents a peak close to 0.8 µm, which resembles the Ceres’ average spectrum. However, it is significantly different from the lizardite spectrum measured at ambient conditions. Along with dehydration caused by the reduction in the water absorption at 3 µm, the high temperature could have significantly dehydroxylated the sample, thus altering its mineralogy and structure. The extent of this alteration deserves further investigation. Here we refer to this sample as “heated lizardite”. 

**Figure 2:**
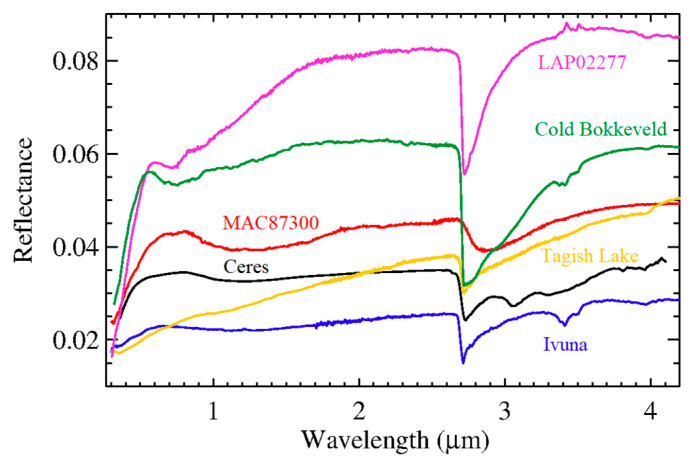
Average Ceres’ reflectance spectrum (black) and reflectance spectra of carbonaceous chondrites (in standard viewing geometry) used to produce the best fit with Ceres’ mineralogy and elemental composition.

Marchi et al. [14] and Kurokawa et al. [15] found that a large fraction of the composition of Ceres should be composed of material similar to carbonaceous chondrites (CC), when taking into account both the IR global average spectrum and the elemental composition from the GRaND measurement. In particular, [14] obtained best fits by adding the CC Ivuna (CI type) or LAP (CM type) to the modeled mixtures; [15] obtained best fits using Ivuna (CI type), Tagish Lake (CM type), or Cold Bokkeveld (CM type). However, our analysis revealed that the above-mentioned CC cannot match both the VIS and IR regions of the average spectrum of Ceres. Instead, we considered MAC 87300 (CM type), which is already mentioned in [2], whose spectral shape is most similar to the Ceres’ spectrum across the two spectral regions (Figure 2).

**Figure 3:**
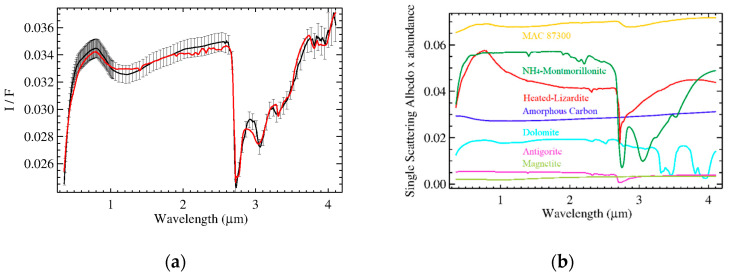
Panel (**a**): measured (black) and modeled (red) average spectrum of Ceres in terms of I/F (r × π). Error bars indicate uncertainties on absolute calibration. The best fit including all endmembers is discussed in De Sanctis et al. [11] with the addition of heated-lizardite (see Table 1), and MAC 87,300 (see Figure 2). In panel (**b**), the single scattering albedo (SSA) of each endmember scaled for their respective relative abundance: MAC 87300 (orange), NH_4_-montmorillonite (dark green), heated lizardite (red), amorphous carbon (blue), dolomite (cyan), antigorite (purple), magnetite (pickle green).

The retrieved mineral abundances are listed in Table 2. Best fit and endmembers scaled for their abundances are shown in Figure 3. Elemental constraints from the GRaND measurements [12,13] (7 wt.% < C < 20 wt.%, H = 1.9 ± 0.2 wt.%, Fe = 16 ± 1 wt.%) are matched by the model (C = 16.5 wt.%, H = 1.8 wt.%, Fe = 16.5 wt.%), according to the densities and elemental compositions of the endmembers reported in [14] used to convert the abundance (vol.%) of minerals retrieved into the elemental composition (wt.%). 

The need for a multiplicative factor and an additional slope (see Table 2) would suggest the presence of additional and/or different endmembers with respect to those assumed: the observed spectrum is darker and redder than the model derived from the endmembers listed in Table 1. However, unknown endmembers should be featureless in the spectral range considered. On the other hand, this discrepancy could also be explained by the effect of space weathering [38].

## 3. Organic-Rich Region

### 3.1. Data Reduction

**Figure 4:**
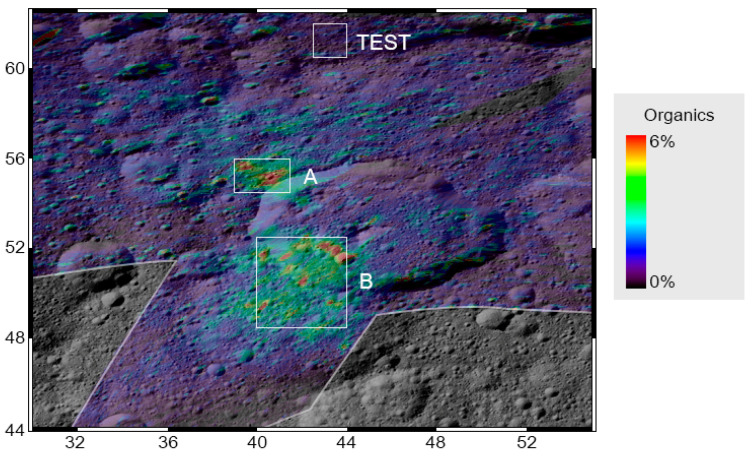
Map of organics’ abundance reported from Raponi et al. [31], projected over a Framing Camera mosaic. The organic-rich areas A and B, and the test area have been marked. Coordinates of the areas are shown in Table 3.

Close to the Ernutet crater (53.4 km diameter, at latitude ~53° N, longitude 45.5° E), a region of ~1000 km^2^ exhibits a prominent and complex 3.4-µm band, composed of several bands: near 3.38–3.39 µm, 3.40–3.42 µm, and 3.49–3.50 µm. These spectral features are characteristic of the symmetric and antisymmetric stretching frequencies of methyl (CH_3_) and methylene (CH_2_). The main candidates for these bands are organic materials containing aliphatic hydrocarbons [18,39], probably with some aromatic hydrocarbons and heteroatoms [34]. Mapping the band depth at 3.4 µm [18,34], the organic material is found mainly in two broad areas: one close to the northwestern portion of the crater, and one located on the southwestern part. Here, we produced two average spectra considering rectangular portions of these two regions (A and B, respectively), delimited by the coordinates shown in Table 3 (Figure 4). Moreover, we accounted for a test area outside the organic-rich regions to rule out any possible instrumental effects that may affect the specific data we collected for the analysis of the organic-rich areas (Figure 4). We accounted for acquisitions in the VIS and IR channel performed simultaneously, which covered the whole area of interest (spacecraft event time: 487085891). For each area, the average spectra obtained from data from the VIS and IR channels were merged to obtain a single spectrum from 0.25 to 5.0 µm. 

**Figure 5:**
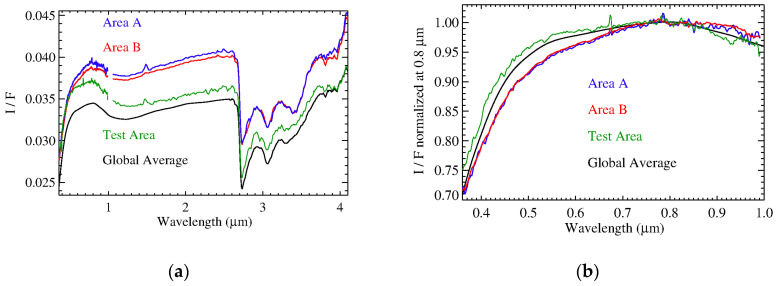
Panel (**a**): Average spectra of area A (blue), B (red) and the test area (green). For comparison, shown is the average Ceres spectrum (black). Panel (**b**): same spectra of panel (**a**) in the VIS range, normalized at 0.8 micron.

Differences between the spectra of the organic-rich areas (A and B) and the test area with respect to the average Ceres spectrum, were highlighted by their normalization (Figure 5). A distinct signature of aliphatic organics is visible at 3.4 microns. Moreover, the absorption at 4 µm shows a larger abundance of carbonates in the organic-rich regions. Such results have already been discussed in [31]. The spectrum of the test area does not present any relevant differences with respect to the average global spectrum, except a slope that is slightly bluer in the visible spectral range. This average test area also samples the typical background over which organic-rich regions stand. As a result, an unusual positive slope (redder) with respect to the global spectrum appears in the visible part of the organic-rich spectra, even more so if we consider the test area spectrum as background. This confirms the previous analysis performed with framing camera data [40]. To interpret this spectral reddening, we performed spectral modeling (Section 3.2), and checked for spatial correlations of the organics with other endmembers (Section 3.3).

### 3.2. Spectral Modeling

We performed spectral modeling following the approach in Section 2.2, using an intimate mixture of the endmembers listed in Table 1.

**Figure 6:**
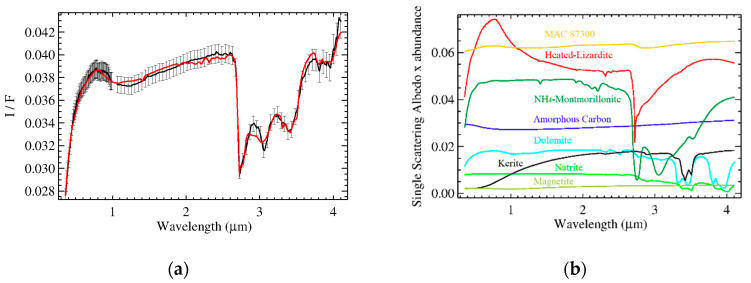
Panels (**a**): measured (black) and modeled (red) average spectrum of Ceres in terms of I/F (r x π) of the organic-rich spectra from area A and area B, respectively. Error bars indicate calibration uncertainties. In panel (**b**), the relative SSA of each endmember scaled for their respective abundance (relative cross section): MAC 87300 (orange), NH4-montmorillonite (dark green), heated lizardite (red), amorphous carbon (blue), dolomite (cyan), antigorite (purple), natrite (light green), magnetite (pickle green), kerite (black).

Spectral fits of the two organic-rich areas are shown in Figure 6. Retrieved abundances and grain size are listed in Table 2. The results are very similar to those discussed in [31], except for the larger content of Mg-phyllosilicates due to the novel introduction of the heated lizardite to match the visible spectral range: the lower depth of the absorption band at 2.7 µm in the spectrum of lizardite with respect to antigorite is compensated by its larger abundance.

As in the case of the global spectrum, a further multiplicative constant and an additional slope are needed to correctly fit the spectra (Table 2).

### 3.3. Spatial Correlations

To attribute the changing slope (Figure 5b) to mineral or organic material requires a comparison of the spatial distribution of the slope in the VIS range with the spatial distribution of compounds. The slope is calculated following Rousseau et al. [17]:

S_0.48–0.80µm_ = [r (0.80 µm) − r (0.48 µm)]/[r (0.48 µm) (λ_0.80µm_ − λ_0.48µm_)]

Being: r (0.80 µm) the median of the reflectance over the range 0.75–0.85 µm (~50 band of the VIS channel), and r (0.48 µm) the median of the reflectance over the range 0.43–0.53 µm (~50 band of the VIS channel).

The mapped abundances of minerals have been reported by Raponi et al. [31], who used the same spectral modeling discussed in Section 2.2, but considering only the IR channel. Thus, the S_0.48–0.80µm_ and the mineral abundances came from two independent datasets.

**Figure 7:**
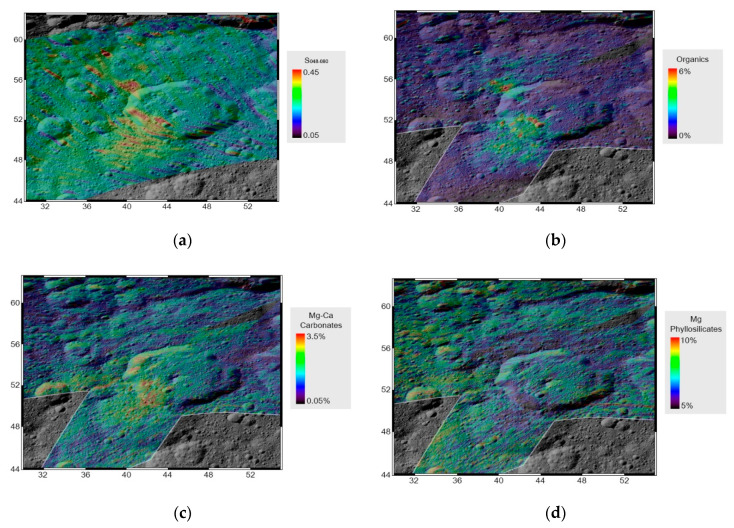
(Panel **a**): projected slope in the range 0.48–0.80 µm. Stripes visible on the maps are artifacts due to the low spatial resolution of the data considered. For comparison, the retrieved abundance of organics (panel **b**), carbonates (panel **c**), and Mg-phyllosilicates (panel **d**) reported from [31].

Comparison of panel (a) and (b) of Figure 7 reveals a spatial correlation between the S_0.48–0.80µm_ and the abundance of organic matter. We also note that other minerals do not present the same degree of spatial correlation. In particular, carbonates are only more abundant in the southern area (B), and Mg-phyllosilicates are mostly anticorrelated. No spatial correlation can be noted for the other minerals (see [31]).

### 3.4. Cerealia Facula 

The most outstanding geological features on Ceres’ surface are the bright areas known as the Ceralia and Vinalia Faculae in the geologically young Occator crater (15.8–24.9° N, 234.3–244.7° E, diameter ~90 km). The faculae have an albedo 5–10 times higher than the average surface [23], with Cerealia Facula being the brightest at the center of the crater. These faculae show distinct spectral differences from the typical crater floor. Absorption at ~3.5 µm and ~4 µm increase toward the brightest areas and are consistent with enrichment in carbonates. A shift of the carbonate band center from 3.95 to 4.01 µm indicates a change in the mineralogy from Mg-carbonate to Na-Carbonate [41]. A complex absorption at 2.20–2.22 µm is observed only in the brightest areas. Raponi et al. [42] have shown that ammonium salts, and more specifically ammonium chloride (NH_4_Cl), provide the best match with this spectral feature. The overall band area between 2.6 µm and 3.7 µm increases from the crater floor to the brightest pixels, and several minima at 2.88, 3.2, 3.28, 3.38, and 3.49 µm emerge and become deeper, revealing the presence of hydrohalite [19]. Moreover, according to spectral modeling, aliphatic carbons, and aromatic hydrocarbons may be present in a small percentage [19]. 

**Figure 8:**
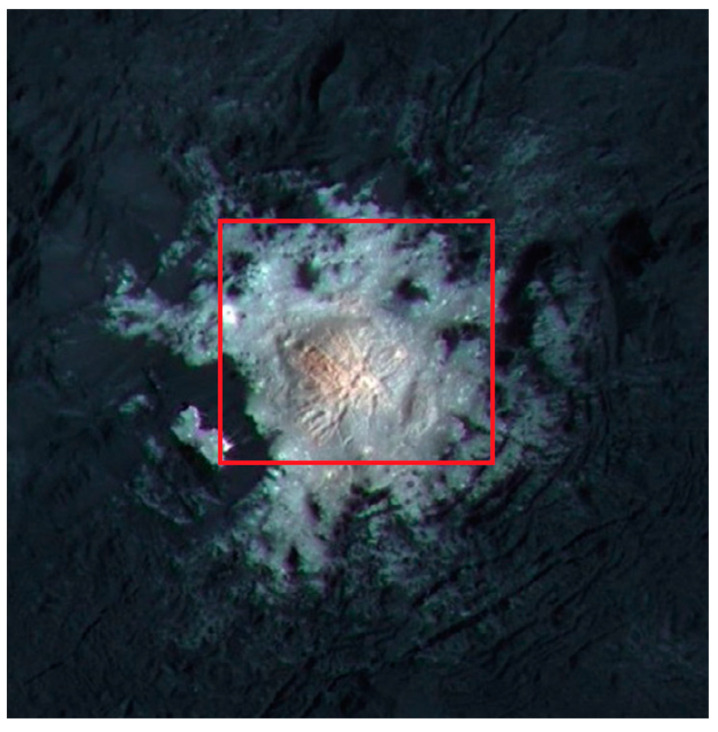
Image of Cerealia Facula obtained by combining framing camera images acquired during LAMO phase with three images using spectral filters centered at 438, 550, and 965 nm. VIR pixel footprints inside the red rectangle (lat: 19.25° N–20.0° N, lon: 239.25° E–240° E) were averaged to obtain the reflectance spectrum [39].

**Figure 9:**
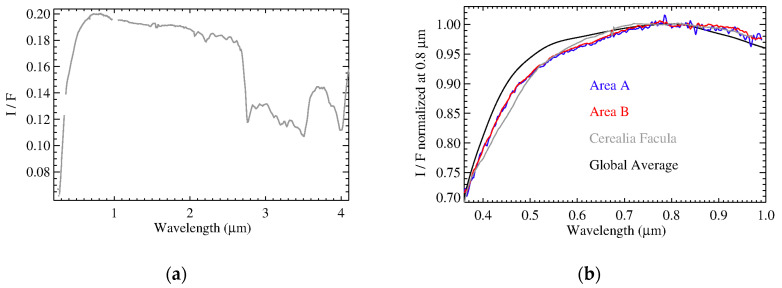
Panel (**a**): Cerealia Facula average I/F spectrum’s VIS and IR channels (gray) were merged to obtain a single spectrum. Spectral range longward of 4.1 µm (not shown here) is affected by a high noise level after the subtraction of the thermal emission. Panel (**b**): focus on the visible spectral range; the spectrum of Cerealia Facula, the global Ceres spectrum, and the organic rich regions (Area A and B) were normalized at 0.8 µm.

The novel possibility of extending the spectral range to the VIS channel can yield new constraints on the mineralogy of the faculae. Here, we took into account an average spectrum of Cerealia Facula, calculated from VIS and IR high spatial resolution data (VIS Spacecraft Event Time: 524703945, IR Spacecraft Event Time: 521436704) that covered the whole region of Cerealia Facula (lat: 19.25° N–20.0° N, lon: 239.25° E–240° E, Figure 8). The spectra in the VIS channel presents a positive slope shortward of 1.0 µm, confirming the previous analysis performed with FC data by Nathues et al. [43]. The red spectrum detected in the VIS range is similar to those observed in the organic-rich spectra coming from the Ernutet regions discussed above (Figure 9). 

## 4. Discussion

### 4.1. Ceres Average Spectrum

The analysis of the average global spectrum of Ceres performed in this study confirms the previous results of Marchi et al. [14], who combined mineralogy (from VIR data) and elemental composition (from GRaND data) to derive Ceres’ average surface composition: the large abundance of carbon, which exceeds that of carbonaceous chondrites, found on a volatile-rich body implies that widespread organic chemistry could have occurred on Ceres. 

The analysis of the average spectrum of Ceres was extended here to the visible part of the spectrum, taking into account the VIS channel of the VIR instrument, which is now available after the setup of the calibration for that channel [16]. The overall shape of Ceres’ spectrum implies that an additional mineral is needed on top of those suggested in De Sanctis et al. [11] and Marchi et al. [14] in order to match the spectral shape in the range 0.3–1.0 µm. Here, we suggested the heated lizardite of Hiroi and Zolensky [35] as a possible analog. Lizardite, like antigorite, is a polymorph of serpentine; however, heating at 600 °C can lead to partial dihydroxylation and structural change, and thus to one or more different silicates. The fit with those thermally processed silicates does not necessarily imply that Ceres’ surface experienced similar temperatures. Instead, it is possible that these silicates are of primary origin, i.e., they were accreted from planetesimals that were thermally processed. Further laboratory measurements are needed to investigate the properties of this silicate, whose applicability to Ceres, if confirmed, would bring new constraints on Ceres’ origin. Moreover, the need for a different carbonaceous chondrite (MAC 87300) than those considered in previous works [14,15], requires further analysis to explain which specific mineral phases determine its spectral shape and match with Ceres’ spectra. 

The spectral modeling presented in this study requires an additional slope and a multiplicative constant, which reddens and darkens the spectrum, respectively, in order to match the observed average spectrum of Ceres. This would compensate for the possible role of space weathering [39]. 

### 4.2. Ernutet Organic-Rich Region

The introduction of the VIS channel data provided new possibilities for refining the modeled composition of the organic rich regions in the Ernutet crater area. Our estimates of organic abundance do not differ significantly from the analysis of De Sanctis et al. [18,34] and Raponi et al. [31]. We point out that the amount of organic material retrieved is strongly dependent on the type of organic that is used for spectral modeling. The limit is represented by the absorption depth of the laboratory spectrum, which cannot be less than the absorption depth measured on Ceres’ surface. Hence, considering different endmembers to match the organic signatures could lead to much larger abundance estimates, e.g., areas matched with 6% of kerite (Figure 7b) would instead require 25% of shocked asphaltite (AS-LXM-004, RELAB) [34]. The retrieved abundance can be even larger (45%–65%) if samples of insoluble organic matter extracted from carbonaceous chondrites are assumed [44]: the less pronounced band at 3.4 µm of the laboratory samples can be compensated by a larger abundance retrieved from the spectral fit. However, we found that kerite provides the best fit with data, both for the shape of the absorption at 3.4 µm and the degree of redness [39]. 

A significant finding obtained from the VIS channel analysis is the detection of an unusual red slope in the VIS range, which has a strong spatial correlation with aliphatic organic abundance. This correlation suggests that this slope is produced by the same organic compound that produces the aliphatic stretching at 3.4 µm, or by a different compound that is closely linked to organics in their formation, conservation, and/or transportation on the surface. 

### 4.3. Cerealia Facula

Sodium salts (NaCl, NaHCO_3_, Na_2_CO_3_, NaCl·H_2_O) identified on Cerealia Facula have also been detected in Enceladus’ plume, suggesting they are sourced from a similar alkaline environment. Furthermore, the presence of hydrated/hydroxylated salts indicates recent exposure of brines, as these compounds are not stable on Ceres’ surface [19]. The current occurrence of brines in Ceres attests to the presence of liquid throughout Ceres’ history. As with Enceladus, one expects that organic chemistry could have been ongoing in Ceres [45,46]. 

The addition of the visible spectral range to the average spectrum of Cerealia Facula adds new constraints on its mineralogy. Here, we highlighted a red slope shortward of 1.0 µm that appears very similar to the absorption over the same wavelength range detected in the Ernutet organic-rich regions. This similarity strongly suggests the presence of organic material in the faculae material, as already suggested by De Sanctis et al. [19]. However, the red slope of the facula with respect the average Ceres terrain could also be attributed to other physical properties of the material, such as different particle size, and/or space weather acting differently with respect to the rest of the surface because of its recent age and unique mineralogy [38]. 

## 5. Conclusions

Based on the amount of data returned by the NASA/Dawn spacecraft and its scientific payload, Ceres is now recognized as a target of astrobiological significance. In particular, Ceres is one of the few bodies where abundant water and organic matter have been found. Here, we reported further indications of an environment in Ceres that is favorable to prebiotic chemistry during the course of its history, and potentially, at present. 

The analysis presented here may open new avenues of investigation on Ceres’ mineralogy, in particular, the attribution of the red slope in the VIS range to organic matter and the nature of the silicates that make up most of Ceres’ regolith (together with the carbon component). Indeed, phyllosilicates can protect organics from space weathering effects because they efficiently absorb organic molecules [47,48]. Moreover, detailed spectral modeling (including the visible range) of the bright material making up the faculae would be desirable in order to improve our knowledge of this hydrated salt-rich mixture and for the information it provides on Ceres’ brine environment. The prospect that organic matter may be present in brines for extended periods of time further supports the value of Ceres as a target of astrobiological significance and calls for follow-up exploration.

## Figures and Tables

**Table 1:** Minerals considered for spectral modeling.

Mineral	Type	RELAB Sample ID/Reference
Antigorite	Mg-phyllosilicate	AT-TXH-007
Heated Lizardite	Mg-phyllosilicate	[35]
NH_4_-montmorillonite	NH4-phyllosilicate	JB-JLB-189
Dolomite	Mg-Ca-Carbonate	CB-EAC-003
Natrite	Na-carbonate	CB-EAC-034-C
Magnetite	Iron oxide	[36]
Kerite	Organic material	MA-ATB-043
Amorphous Carbon	Organic material	[37]

**Table 2:** Retrieved abundance and grain size of endmembers. In brackets the values retrieved for Area B have been reported when different from those of Area A.

Mineral	Area A (Area B)	Global Average
MAC 87300	56%	60%
Heated Lizardite	12%	10%
Antigorite	1%	1%
NH_4_-montmorillonite	5%	6%
Dolomite	2%	2%
Natrite	1%	0%
Magnetite	1%	1%
Kerite	2%	0%
Amorphous Carbon	20%	20%
Grain size	270 µm	210 µm
Multiplicative factor	0.72 (0.74)	0.63
Additional Slope	7.8 (7.6) 10^−3^ µm^−1^	7.8 10^−3^ µm^−1^

**Table 3:** Range of coordinates of two rectangular areas inside the organic-rich regions, and a test area. The number of spatial pixels inside the areas used to perform the average spectra is provided.

Area	Lon (°)	Lat (°)	N. Pixel VIS	N. Pixel IR
A	39.0–41.5	54.5–56.	22	22
B	40.0–44.0	48.5–52.5	184	180
Test	42.5–44.0	60.5–62.0	127	145
